# Medication documentation in a primary care network serving North Carolina medicaid patients: results of a cross-sectional chart review

**DOI:** 10.1186/1471-2296-13-83

**Published:** 2012-08-13

**Authors:** Matthew D Olson, Gretchen L Tong, Beat D Steiner, Anthony J Viera, Evan Ashkin, Warren P Newton

**Affiliations:** 1 Marshfield Clinic – Lake Hallie Center, Department of Family Practice, , 12961 27th Avenue, Chippewa Falls, WI, 54729, USA; 2 Department of Family Medicine, University of North Carolina School of Medicine, CB #7595, Chapel Hill, NC 27599, USA

**Keywords:** Congruence, Medication documentation, Medical record, Patient safety, Medication discrepancy, Community Care of North Carolina, Community network

## Abstract

**Background:**

Medical records that do not accurately reflect the patient’s current medication list are an open invitation to errors and may compromise patient safety.

**Methods:**

This cross-sectional study compares primary care provider (PCP) medication lists and pharmacy claims for 100 patients seen in 8 primary care practices and examines the association of congruence with demographic, clinical, and practice characteristics. Medication list congruence was measured as agreement of pharmacy claims with the entire PCP chart, including current medication list, visit notes, and correspondence sections.

**Results:**

Congruence between pharmacy claims and the PCP chart was 65%. Congruence was associated with large chronic disease burden, frequent PCP visits, group practice, and patient age ≥45 years.

**Conclusion:**

Agreement of medication lists between the PCP chart and pharmacy records is low. Medication documentation was more accurate among patients who have more chronic conditions, those who have frequent PCP visits, those whose practice has multiple providers, and those at least 45 years of age. Improved congruence among patients with multiple chronic conditions and in group practices may reflect more frequent visits and reviews by providers.

## Background

In its widely cited 2001 report, *Crossing the Quality Chasm*, the Institute of Medicine (IOM) stated that most medical records are disorganized, illegible, and inaccessible, “making it nearly impossible to manage many forms of chronic illness that require frequent monitoring and ongoing patient support [1].” Furthermore, medication errors are the cause of substantial mortality in both the inpatient and outpatient settings; in *To Err is Human*, the IOM estimated that 1 in 131 outpatient deaths and 1 in 854 inpatient deaths were caused by medication errors [2]. In 2001, the IOM included safety among its “Six Aims for Improvement,” emphasizing that patient information be accessible and available “to all who need to know it [1].” Accurate charting of patient medications is crucial to establishing the safe medical system envisioned by the IOM.

Because the medical chart of the primary care provider (PCP) is a critical component of maintaining quality care, medication documentation errors in the PCP chart may present a risk for medication errors [3,4]. However, our knowledge of the extent and characteristics of medication documentation errors in outpatient settings is limited. The research conducted to date have been small studies that focus on single practices [5,6], nursing homes [7], hospital services [8-10], inclusion of limited classes of medication(s) [11-17], inclusion of specific patient populations (geriatric patients [18,19]), or rely on phone [20,21] to gather patient information.

This study was performed to expand current understanding of the problem of medication documentation in outpatient settings, by documenting the frequency of prescribed medication congruence in representative practices providing primary care, including private offices, community health centers (CHC) and academic health center affiliated clinics. A secondary goal was to identify practice and patient characteristics associated with better medication documentation in order to inform future interventions to decrease adverse outcomes from medication errors. Because of the limited sample size, the results of this study should be considered for hypothesis generating only.

## Methods

### Source of study participants

Subjects were drawn from practices that belong to one Community Care of North Carolina (CCNC) network. CCNC has been described in detail elsewhere [22,23], but briefly, it is a statewide community health network of approximately 1200 primary care practices that manages care for 1,000,000 Medicaid patients. One of these networks, of 50 local practices, was selected as a source for study participants because it allowed easy access to complete record of all paid medication claims information through the CCNC pharmacy home program.

A convenience sample of eight practices in the network were selected to represent a spectrum of characteristics to be studied: practice type (academic, private, CHC), practice size (solo, 2-5 providers, >5 providers), and type of medical record (paper, electronic). Patients at high-risk for polypharmacy, those with at least 15 medication fills in the previous 90 days or visit claims from at least 3 practices in the previous 6 months, were selected for study as it was hypothesized that they would be at greater risk for medication errors, including the lack of congruence. The 8 practices identified had 421 patients eligible for the study. Based on a standard deviation of 10%, an alpha of 5%, and 80% power, a 10% difference in congruence between 2 groups could be identified with a sample size of 32 patients; however a larger sample size was used because no pilot data were available on which to base the estimated standard deviation. Eligible patients from these practices were arranged in random order for each practice using a computerized randomization algorithm, and the first 13 patients from each practice were selected to achieve a sample size of 104. Fewer than 13 eligible patients were available for some practices, so additional patients were selected sequentially from a practice with similar characteristics, yielding a final sample size of 100, with an average of 12.5 patients from each practice (range 11-15).

### Measuring agreement between chart and patient: medication congruence

A chart review was performed between November 9 and December 20, 2007 to obtain the PCP medication list. Medications were abstracted from the PCP chart if they were documented in the current medication list, visit notes, or correspondence from the previous 12 months.

We used pharmacy records as a proxy for the medications that the patient has taken in the previous year. Apart from direct observation, it is difficult to obtain a fully accurate measure of medications that the patient is taking but previous studies have shown that medication claims are a good approximation of medication consumed [19,24]. Access to all pharmacy claims paid to all pharmacies for the CCNC patients included in this study were obtained for the 365 days immediately preceding the chart review. There were no known programs or discounts that would have provided patients with lower costs than their co-pay. Therefore, it is felt that the pharmacy claims would represent the medications obtained by the patient.

**Figure 1:**
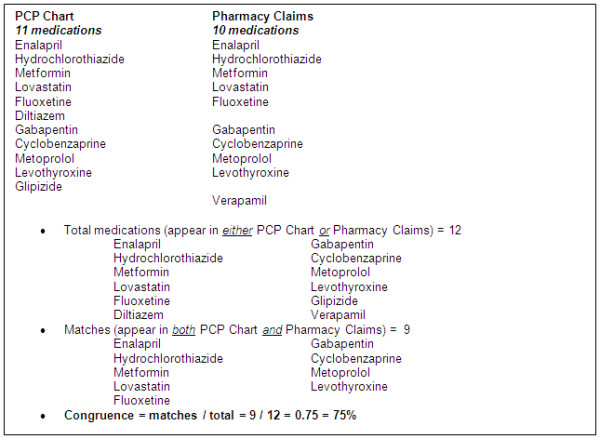
Sample congruence calculation.

Medications were excluded from both the PCP and claims list if they were over-the-counter (OTC), as needed (PRN), or medications for short-term or acute conditions (e.g., antibiotics). If the medication appeared in the PCP chart but not the pharmacy claims, and if there was an OTC form available, the medication was considered OTC. Medications that may be obtained OTC were included if they appeared in the pharmacy claims. Medications were considered PRN only if explicitly written as such in the PCP chart, or if standard administration of the medication is on a PRN basis. A medication was considered short-term or acute if the chart specifically indicated it was to be used for a limited time or if it appeared in the pharmacy claims but did not have at least 1 fill with a minimum 28-day supply. Because dose, route, and frequency are not provided by the pharmacy claims, matching was only on active ingredient of the agent. The term congruence has been used to describe the degree to which medication lists are in agreement with each other [25-28]. Congruence—the percent agreement between 2 medication lists—provides an important indicator of errors of inclusion or omission in a medication list. In this study, pharmacy claims serve as the proxy measure of what the patient has taken in the previous year while the list of all medications found in the PCP chart—including current medication list, visit notes, and correspondence—serves as a proxy for the PCP medication list for the previous year. Congruence in this study is the agreement between these two lists. A sample calculation of congruence is included in Figure 1. 

### Other variables

To study factors associated with medication congruence, a number of variables were abstracted. Patient age at time of chart review and sex were obtained from the CCNC patient database. Number of documented chronic conditions was extracted from the chart from the current problem list, visit notes and correspondence from the previous 12 months. The documented presence of 3 diagnoses commonly encountered in primary care—hypertension, diabetes, and mental illness (including substance abuse)—was identified for each patient, as were number of PCP visits in the past year, date of last PCP visit, number of providers in practice (including physicians, physician assistants, and nurse practitioners), type of practice (academic, private, or CHC), and type of medical record (paper or electronic). Patients with 8 or more chronic conditions documented were designated as having a large chronic disease burden, and patients making 9 or more visits to their PCP in the past year were categorized as having frequent PCP visits.

### Statistical analysis

We examined the percent congruence by demographic, clinical, and practice characteristics and tested for differences using a two-sample t-test (for dichotomous variables) or one-way ANOVA (for variables with three categories). For practice-level characteristics, analyses were adjusted for clustering by practice. For this study, 2-sided p-value ≤ 0.05 was considered significant. Statistical analysis was performed using Stata/IC 10.0 for Windows (StataCorp LP, College Station, TX).

### Human subjects review

Approval was obtained from the University of North Carolina Biomedical Institutional Review Board.

## Results

### Pharmacy claims and PCP medication list

The average number of medications in pharmacy claims data was 10 per patient (Table 1), and the medication list obtained from thorough chart review was also an average of 10 medications per patient.

**Table 1:** Patient characteristics (n = 100)

	**Mean (SD, range), or Percent**
Age, mean (SD, range)	46 (12, 19-81)
Female sex, %	74%
# of PCP visits past 12 months, mean (SD, range)	7 (5, 0-25)
Days since last PCP visit, mean (SD, range)	95 (153, 0-1058)
Number of documented chronic conditions in PCP chart, mean (SD, range)	6 (3, 0-13)
Common diagnoses	
Mental illness (including substance abuse), %	70%
Diabetes, %	35%
Hypertension, %	50%
Medications in PCP chart (medication list, visit notes, correspondence), mean (SD, range)	10 (5, 1-26)
Medications in pharmacy claims, mean (SD, range)	10 (5, 1-27)
Total medications in chart and claims, mean (SD, range)	12 (6, 1-28)
Type of practice patient visited, %	
Community Health Center	24%
Academic Practice	24%
Private Practice	52%
Number of providers in practice patient visited, %	
1	13%
2 or more	87%
Medical record type of practice patient visited, %	
Electronic	48%
Paper	52%

### Patient characteristics

Patient age, number of PCP visits in the past year, days since last PCP visit, number of chronic conditions, and percentage with three common primary care diagnoses are reported in Table 1.

### Practice characteristics

Type of practice, practice size, and practice record type are reported in Table 1.

### Overall congruence

Overall congruence showed an average 65% agreement of active medication ingredient name (SD 22%) (Table 2). The range for congruence was 0-100%, with 2 of the 100 charts having no agreement with pharmacy claims and 8 charts having perfect agreement.

**Table 2:** Medication congruence

	**Congruence (SD)**
Congruence = percent agreement between pharmacy claims and medications found in current medication list, visit notes, or correspondence sections of PCP chart	65% (22%)

### Factors associated with congruence

Greater congruence was significantly associated with older age, larger chronic disease burden, more frequent PCP visits, and group practice (Table 3).

**Table 3:** Prescribed medication list congruence by demographic, clinical, and practice characteristics

	**Congruence**	**p-value**
Age		
<45 years	59%	0.02
≥45 years	69%	
Sex		
Female	65%	0.56
Male	63%	
Mental illness (including substance abuse)		
Yes	66%	0.26
No	62%	
Hypertension		
Yes	68%	0.10
No	61%	
Diabetes		
Yes	69%	0.14
No	62%	
Large documented chronic disease burden		
Yes	72%	0.04
No	62%	
Frequent PCP visits in past year		
Yes	72%	0.04
No	62%	
Medical record*		
Electronic	66%	0.69
Paper	64%	
Type of practice*		
Community Health Center	66%	0.68
Academic Practice	65%	
Private Practice	64%	
Number of providers in practice*		
1	58%	0.02
2 or more	66%	

*Practice-level characteristics adjusted for clustering by practice.

## Discussion

This study demonstrates poor medication list congruence between the primary medical chart and patient (as measured by filled prescriptions), and even a thorough review of the chart yielded only 65% congruence with pharmacy claims.

These results extend findings of a 2001 study that found 65% mean congruence between patient medication lists and primary physician charts in a single academic primary care practice [25]. This study differed from ours in that the patient medication list was obtained during a home visit, and the PCP extracted the medication list from the entire chart. This method of chart extraction is similar to ours, and their findings are comparable to our congruence of 65%.

This study should serve as a wake up call. We found that approximately one third of the medications listed in either a PCP’s chart or that a patient is taking do not match between these two lists. Even more impressively, exact matches of medication names from the two sources were only found for 8% of the charts reviewed.

We found higher congruence among patients seen in offices with more than one provider. It is possible that interaction among providers results in more rapid diffusion of new ideas and thus potentially greater readiness to accept recent pushes towards quality improvement. Larger practices also have to rely more heavily on effective communication between providers perhaps making clear medical records and efforts to update the medication list a higher priority.

Interestingly, older age, frequent PCP visits, and larger documented chronic disease burden were associated with higher congruence. It is possible that these patients, despite having more complicated medication lists, are reviewed more carefully and more frequently and thus have more accurate lists or that increased documentation of chronic disease burden is correlated with more accurate documentation of medication lists.

This study has some important limitations. Most important, pharmacy claims data provided only medication name and dosage form—thorough medication documentation should include dose, route, and frequency. Because it was only possible to match on medication name, it is likely that our estimates of medication congruence are overly optimistic. However, using pharmacy claims data as a proxy for patient medication list avoided some sources of error—especially recall bias that may be associated with patient interviews. In addition, matching only on pharmacy claims precluded measurement of clinically important OTC medications, most notably aspirin. Furthermore, it was rarely possible to ascertain from the PCP chart exact start and stop dates for prescriptions, so a medication no longer being taken by the patient (with or without the PCP’s knowledge) but still in the PCP chart would have counted as a congruent medication. This limitation may, in effect, overestimate congruence; though we feel this limitation is mitigated by our use of a full year of pharmacy claims compared to a full year review of the PCP chart. As our patient population included patients with a higher number of medication fills or practices visited, this may limit generalizability to patients with less medications and less providers. In addition, this study had a relatively small sample size and was limited to a small geographic location. Finally, in keeping with this being an exploratory study, only bivariate analyses were performed of predictors of congruence, and results were not adjusted for the number of medications.

Because of these limitations, our findings are useful primarily for hypothesis generating and need confirmation with larger studies. Future studies should focus on establishing causal relationships between medication congruence and the associated factors we identified. Such studies could employ similar network-wide samples as ours, but use larger samples and state a priori hypotheses. For example, because the PCP medical record is valuable in establishing medication orders on hospital admission and because the PCP medical record may fall out of synch with patient medications after hospital discharge, understanding the effect of hospitalization on outpatient medication congruence would be a valuable future study. More broadly, it will be interesting to see if patient managed electronic medical profiles can be used as a more reliable source of accurate medication lists.

## Conclusions

The wider implications of this study are clear. First, the primary medical record of many patients is deficient, which may be an open invitation to medical errors. Also, our findings do not support the hypothesis that complex patients and poor medical records go hand in hand—indeed, increasing number of chronic conditions was associated with improved medication congruence.

## Abbreviations

EHR: Electronic health record; IOM: Institute of Medicine; CCNC: Community care of North Carolina; CHC: Community Health Center; PCP: Primary care provider; OTC: Over the counter; PRN: As needed.

## Competing interests

Matthew D. Olson, MD, MPH - declares no potential, perceived, or real conflicts of interest.

Gretchen L. Tong, PharmD - declares no potential, perceived, or real conflicts of interest.

Beat D. Steiner, MD, MPH - declares no potential, perceived, or real conflicts of interest.

Anthony J. Viera, MD, MPH - declares no potential, perceived, or real conflicts of interest.

Evan Ashkin, MD - declares no potential, perceived, or real conflicts of interest.

Warren P. Newton, MD, MPH - declares no potential, perceived, or real conflicts of interest.

## Authors’ contributions

MO participated in the design of the study, performed data collection, organization, and statistical analyses, drafted the manuscript, and participated in manuscript revising. GT participated in the design of the study, its coordination, and manuscript revisions. BS conceived of the study, participated in its design and manuscript revising. AV advised on and participated in statistical analyses and manuscript drafting. EA conceived of the study, participated in study design, and manuscript revising. WN participated in study conception and design and contributed to manuscript content. All authors approved the final manuscript.

## Authors’ information

MO – The Marshfield Clinic – Lake Hallie Center, Department of Family Practice.

GT, BS, AV, EA, WN – University of North Carolina School of Medicine, Department of Family Medicine.

GT – Regional Pharmacist for AccessCare, a Community Care of North Carolina network.

## Pre-publication history

The pre-publication history for this paper can be accessed here:

http://www.biomedcentral.com/1471-2296/13/83/prepub

## Author details

^1^Marshfield Clinic – Lake Hallie Center, Department of Family Practice, , 12961 27th Avenue, Chippewa Falls, WI 54729, USA. ^2^Department of Family Medicine, University of North Carolina School of Medicine, CB #7595, Chapel Hill, NC 27599, USA.
